# Observed Normativity and Deviance in Friendship Dyads’ Conversations About Sex and the Relations With Youths’ Perceived Sexual Peer Norms

**DOI:** 10.1007/s10508-016-0763-x

**Published:** 2016-07-08

**Authors:** Daphne van de Bongardt, Ellen Reitz, Geertjan Overbeek, Marie-Aude Boislard, Bill Burk, Maja Deković

**Affiliations:** 1 Research Institute of Child Development and Education (Research Priority Area YIELD), Faculty of Social and Behavioural Sciences, University of Amsterdam, P.O. Box 15776, 1001 NG Amsterdam, The Netherlands; 2 Utrecht Centre for Child and Adolescent Studies, Faculty of Social and Behavioural Sciences, Utrecht University, Utrecht, The Netherlands; 3 Sexology Department, Faculty of Human Sciences, Université du Québec à Montréal, Montreal, Canada; 4 Behavioural Science Institute, Faculty of Social Sciences, Radboud University Nijmegen, Nijmegen, The Netherlands

**Keywords:** Adolescents, Friendships, Peer norms, Sexual behavior, Actor–partner interdependence model (APIM)

## Abstract

The current study examined the relations between observed normativity and deviance during adolescents’ and young adults’ conversations about sex with their friends and their individual perceptions of sexual peer norms. Participants were 16–21-year-old same-sex friendship dyads (31 male and 30 female dyads) who performed a peer interaction task that consisted of five discussion assignments focusing on party planning, sexual double standards, condom use, homosexuality, and consensual sex. Videotaped discussions were coded to capture the amounts of normative talk (e.g., consistent with notions of healthy sexuality) and deviant talk (e.g., consistent with notions of risky sexuality), and the verbal or nonverbal reinforcement thereof. Participants also completed individual questionnaires to assess their perceived sexual descriptive norms, injunctive norms, pressure, and risk norms among their peers. Actor–partner interdependence model (APIM) results revealed that youths’ perceived descriptive, injunctive, and risk norms, but not their experienced peer pressure, were related to both their own (actor effects) and their friends’ (partner effects) normativity and deviance. Overall, more deviance was related to perceiving friends to be more sexually active, more approving of having sex, and engaging in more risky sex, whereas more normativity was related to these perceptions in the opposite direction. Gender differences in the APIMs indicated that interactive normativity and deviance was related to perceived descriptive, injunctive, and risk norms for boys, but only to perceived injunctive norms for girls. These findings demonstrate the importance of assessing the dyadic nature of youths’ sexual communication with friends, their relation to individual sexual peer norm perceptions, and gender differences therein.

## Introduction

The developmental periods of adolescence and young adulthood are characterized by significant changes in various domains (Arnett, 2000; Lerner & Galambos, 1998; Steinberg & Morris, 2001). Two of the most prominent changes concern the increasing intensity and importance of relations with peers, and the expanding exploration of sexual behaviors. Regarding the latter, in many Western countries, half of the adolescents have engaged in intercourse before they turn 18, with median ages being 17.0 years for the UK (Johnson et al., 2012), 17.1 years for the Netherlands (De Graaf, Kruijer, Van Acker, & Meijer, 2012), and 17.4 years for the U.S. (Finer, 2007). Besides the normative developmental task of increasing engagement in sexual behaviors (Tolman & McClelland, 2011), from early adolescence to young adulthood, the frequency of interactions with peers typically increases (Larson & Richards, 1991; Richards, Crowe, Larson, & Swarr, 1998), as does the importance of peer feedback for youth’s self-evaluation and identity formation (Hergovich, Sirsch, & Felinger, 2002; Parker, Rubin, Erath, Wojslawowicz, & Buskirk, 2006). Hence, peers become crucial sources of emotional support, and significant social referents for behavioral decisions, including those that are related to the engagement in sexual behaviors.

According to social learning theory (Bandura, 1971), youths’ engagement in new behaviors is stimulated by their observations of the behaviors engaged in by peers. This process of observational learning is based on the reasoning that if others do it, it is probably a good or wise thing to do (Fekadu & Kraft, 2002; Rivis & Sheeran, 2003). Imitation of peer behaviors can be intrinsically rewarding, as it contributes to a favorable sense of self. However, the imitation of behaviors that peers engage in can also be extrinsically motivated, for instance, through social rewards and punishments, such as inclusion or exclusion, or higher or lower status (Brechwald & Prinstein, 2011; Cialdini & Trost, 1998). Observational learning has been proposed as an important explanation for the fact that adolescents’ and young adults’ sexual behaviors and attitudes toward sexuality are often very similar to those of their friends. However, according to social norm theory (Cialdini & Trost, 1998), these “homophily effects” (Brechwald & Prinstein, 2011; Kandel, 1978) can not only be explained by observing and imitating actual behaviors of peers, but are related to youths’ perceptions of which behaviors are prevalent, accepted, or desired among peers, altogether referred to as “social norms” (Cialdini & Trost, 1998). A recent meta-analysis has shown that three types of sexuality-related social norms among peers can be distinguished: descriptive norms, injunctive norms, and peer pressure (van de Bongardt, Reitz, Sandfort, & Deković, 2015). Descriptive norms refer to actual or perceived sexual behaviors of peers, and can relate to peers’ overall sexual activity or their engagement in risky sexual behavior (hereafter referred to as “risk norms”). Injunctive norms refer to actual or perceived attitudes (i.e., approval or disapproval) of peers regarding the engagement in sexual behavior. Peer pressure refers to the active and explicit encouragement from peers to engage in sexual behavior.

In their meta-analysis, van de Bongardt et al. (2015) found that all three types of sexual peer norms were related to adolescent sexual behavior. Adolescents who perceived their peers as more sexually active, more approving of having sex, and as exerting more pressure on them to have sex, tended to be more sexually active themselves. Similarly, adolescents who believed that their peers engaged in more risky sexual behavior were more likely to engage in such behavior themselves. However, even though their relations with adolescent sexual behavior have been confirmed, not much is known about how these different types of sexual peer norms are established, and how adolescents and young adults come to perceive such norms. With the current study, we aimed to shed new light on this by examining how communication with peers about sexuality-related topics (hereafter referred to as “sexual communication”) might be related to adolescents’ and young adults’ perceptions of existing sexual norms among peers.

In doing so, we build on research that suggests that communication may play an important role in adolescents’ perceptions of sexual peer norms. An American study that was conducted among 316 adolescents between the ages of 14–16 years showed that girls, but not boys, who communicated more frequently with friends about sex experienced more pressure (a combination of descriptive and injunctive norms) to have sex (Busse, Fishbein, Bleakley, & Hennessy, 2010). However, although this study has demonstrated a link between sexual communication with friends and adolescents’ perceptions of sexual peer norms, it only assessed adolescents’ self-reported frequency of sexual communication with friends, but not *how* they talked about these topics. Yet, it might be exactly the way in which youths talk about sex with friends, rather than how often they discuss it, that can explain how peers affect youths’ sexual peer norm perceptions and their sexual decision making. For instance, adolescents and young adults who discuss sexuality more normatively with their friends (i.e., in line with notions of healthy and pleasurable sexuality) may be more inclined to internalize these notions as their own norms, and may thus be more likely to engage in responsible and healthy sexual behaviors. In contrast, youths who tend to engage in a more deviant sexual discourse with their peers (i.e., in line with notions of risky or nonconsensual sexuality), may be more disposed to internalize such notions as their own norms, and may thus be more likely to engage in risky sexual behavior.

Evidence for the importance of distinguishing between a more normative versus a more deviant character of communication between peers comes from a significant body of research on processes of deviancy training (i.e., rule-breaking talk and reinforcement thereof) during peer interactions in micro-time (i.e., over the course of seconds, minutes, or hours) and the link with the development of problem behaviors in macro-time (i.e., across years; Patterson, Dishion, & Yoerger, 2000; Wachs, 2015). Various studies have shown that the amounts of observed rule-breaking versus normative talk, and the reinforcement thereof, during videotaped interactions between adolescents and their friends was associated with the development of problem behaviors, including antisocial tendencies, aggression and violence, substance use, and risk-taking (e.g., Dishion, Capaldi, Spracklen, & Li, 1995; Dishion, Eddy, Haas, Li, & Spracklen, 1997; Patterson et al., 2000; Piehler & Dishion, 2007). More specifically related to sexuality, Capaldi, Dishion, Stoolmiller, and Yoerger (2001) examined the relation between the contents of observed conversations between 17–18-year-old male adolescents and their friends, and physical and psychological aggression toward female partners. As one segment of the videotaped peer-interaction task, the 206 participants were instructed to talk for 5 min about what they liked and disliked about the girls they knew. It was found that observed hostile talk about women with male peers was related to later aggression toward a female partner.

Together, these observational studies have demonstrated that the way in which youths interact and talk with their friends is related to their own behaviors. Building on research that demonstrated sexual communication with friends to be associated with adolescents’ perceptions of sexual peer norms (Busse et al., 2010), we propose that this link might be explained by the social norms that adolescents and young adults interactively construct, display, and observe through normative and deviant talk with their friends, which in turn have been found to guide their own attitudes and behavioral decision making (van de Bongardt et al., 2015). Hence, the goal of the current study was to investigate the relation between the amounts of normativity and deviance during sexual communication among the16–21-year-old friendship dyads and individual dyad members’ perceptions of four types of sexual peer norms (i.e., descriptive, injunctive, and risk norms, and peer pressure). Moreover, we aimed to extend the existing literature in two ways.

First, considering the dyadic nature of peer interactions, we examined how adolescents and young adults within each dyad mutually affected each other’s perceptions of sexual peer norms, by using actor–partner interdependence modeling (APIM) (Olsen & Kenny, 2006). In APIM, it is assumed that each dyad member’s own sexual communication scores affect both his/her own perceived sexual peer norm scores (i.e., actor effects) and his/her friend’s perceived sexual peer norm scores (i.e., partner effects). Thus, in this model, both dyad members are considered as a target as well as a source of normative and deviant sexual communication. By using APIM, we aimed to extend observation studies that typically select one dyad member for the analyses (e.g., Capaldi et al., 2001; Dishion, Nelson, Winter, & Bullock, 2004; Patterson et al., 2000; Piehler & Dishion, 2007), as this approach better resembles the interactive processes of mutual influence that play a role during peer interactions.

Second, whereas observation studies typically include male dyads only (for two exceptions, see Dishion, 2000; Piehler & Dishion, 2007), in the current study, we observed both male and female dyads. As such, we were able to assess gender differences in the levels of normativity and deviance during sexual communication with friends, in the perceptions of sexual peer norms, and in the relation between these two (i.e., in the APIM models). In observation studies that investigated peer deviancy training among both boys and girls, female dyads engaged in deviant talk less often, and were rated as more mutual in the type of talk (e.g., normative or deviant) than male dyads (Dishion, 2000; Piehler & Dishion, 2007), which stresses the importance of assessing gender differences in dyadic peer interactions.

Together, these aims lead to the following hypotheses. First, we hypothesized that more normative sexual communication with friends, and reinforcement thereof, would be related to youths’ individual perceptions of less sexual activity among peers, less approval from peers to have sex, less pressure from peers to have sex, and less risky sexual behavior among peers (*H1a*). In contrast, we expected that more deviant sexual communication with friends, and reinforcement thereof, would be associated with perceptions of more sexually active peers, more sex-approving peers, more peer pressure to have sex, and more risky sexual behavior among peers (*H1b*). Regarding the dyadic nature of the observed peer interactions, we expected that youths’ own normativity and deviance (actor effects) as well as their friend’s normativity and deviance (partner effects) during the observed peer interactions would be related to their individual perceptions of sexual peer norms (*H2*). Regarding gender differences, we anticipated female dyads to engage in more normative sexual communication, and male dyads to display more deviant sexual communication (*H3a*). Furthermore, we expected that boys would overall perceive more sex-promoting peer norms than girls (*H3b*), in line with the sexual double standard (Crawford & Popp, 2003; Lyons, Giordano, Manning, & Longmore, 2011). Our expectations regarding gender differences for the APIM analyses (*H3c*) were less straightforward. On the one hand, we assumed that peer interactions, particularly partner effects, may matter more for girls, who are often found to be more sensitive to social influences than boys (Cialdini & Trost, 1998; Rudolph & Conley, 2005). On the other hand, it was possible that effects of sexual communication with friends, particularly partner effects, would be stronger for boys, who generally experience more pressure from peers to have sex (Brown, Clasen, & Eicher, 1986), and for whom sexual activity is more important for their same-sex peer acceptance (Kreager & Staff, 2009; Reed & Weinberg, 1984). As neither theory nor research provided definitive hypotheses about gender differences in the investigated relations, these analyses had an exploratory character.

Notably, at the age of the current study sample sexual behavior can be considered normative (De Graaf et al., 2012; Tolman & McClelland, 2011), and thus perceiving friends as sexually active (i.e., descriptive norms) or as approving thereof (i.e., injunctive norms) would not necessarily be problematic at this age. Nonetheless, investigating the hypothesized associations is still highly relevant as these types of peer influence may become problematic in the case of more extreme sexual norms or behaviors among peers, as they may be particularly problematic for younger adolescents, and as the other types of peer influence (i.e., peer pressure and perceived risk norms) can be considered potentially problematic for youths of all ages.

## Method

### Participants

Data for the current study were collected as part of a larger study, “Project STARS” (Studies on Trajectories of Adolescent Relationships and Sexuality), which was conducted in the Netherlands, with approval from the ethics board of the Faculty of Social and Behavioural Sciences of Utrecht University. A total of 122 adolescents and young adults (62 males and 60 females) between 16 and 21 years (*M* = 17.3 years, *SD* = 0.94) participated in the current observation study. The majority of the participants had a Dutch or other Western ethnic background, and 18.9 % had a non-Western ethnic background. About two-thirds (63.9 %) had a high education level (i.e., senior general education or pre-university education), and about one-third (36.1 %) had a low educational level (i.e., pre-vocational education).

### Procedure

The participants were recruited through sports clubs, youth venues, malls, and personal networks of research assistants. Recruitment took place in large cities and small municipalities in different areas of the Netherlands. Eligible youths were asked to participate in the study with a same-sex friend. Both dyad members had to be between 16 and 21 years of age. This resulted in 31 male and 30 female participating friendship dyads. Most participants (94.3 %) described each other as a good or best friend.

The observations took place at locations that were preferred by the participants and where maximum privacy was ensured (e.g., the participants’ home, school, etc.). All observations were guided by trained research assistants (undergraduate and graduate students from Utrecht University). Before the observations started, the research assistants explained the goals and procedure of the study, guaranteed confidentiality, and pointed out the option to withdraw participation at any time. The research assistants were not present during the assignments, but kept track of time outside of the observation space, in order to ensure maximum privacy, and to stimulate the dyads to talk freely (Mathys, Hyde, Shaw, & Born, 2013; Piehler & Dishion, 2007). After completing all assignments, each participant received a movie coupon (€10).

Following the observations, participants were asked to complete an individual online questionnaire, which yielded information about their perceptions of sexual peer norms. After completing the questionnaire, participants received an additional book certificate (€5). Of the total observation sample, 80 participants (65.6 %) subsequently completed the online questionnaire (on average 1.4 months, *SD* = 1.8, after the observations). The questionnaire subsample did not differ from the total observation sample on demographic characteristics (i.e., gender, age, ethnicity, or education level), indicating that the subsample that completed the online questionnaire consisted of a proper representation of the total observation sample. However, in the questionnaire subsample, more participants reported having had sex (75.0 %) than in the total sample (54.8 %), *χ* ^*2*^(1) = 5.19, *p* = .020. We chose to retain the whole sample for the analyses and deal with the missing values for the sexual peer norm variables of the participants who did not complete the online questionnaire, because it has been shown that this yields more accurate results than listwise deletion, even when data are not missing completely at random (Schafer & Graham, 2002).

### Measures

During the observations, the participating friendship dyads performed an adapted version of the Peer Interaction Task (PIT) (e.g., Dishion, Andrews, & Crosby, 1995; Dishion, Spracklen, Andrews, & Patterson, 1996; Patterson et al., 2000; Piehler & Dishion, 2007), which was specifically designed to stimulate participants to discuss sexuality-related topics. The resulting dyad interactions, which lasted approximately 30 min, were videotaped. The interaction task included five assignments: (1) party planning, (2) boys versus girls, (3) condom use, (4) homosexuality, and (5) boundaries and wishes (5 min each; see Table 1).^1^ Although the first assignment was designed as a warming-up activity, this assignment also triggered sexual communication, and was therefore included in the analyses. Assignments 2–5 were based on the content of questionnaire instruments that have been used in large-scale studies on adolescent sexual development in the Netherlands (De Graaf et al., 2012; Deković, Van Aken, Ter Bogt, & Van Geert, 2010), and which have been shown to reliably and validly measure adolescents’ and young adults’ sexuality-related attitudes and values. The participants were instructed to read each assignment out loud, and to discuss out loud what they thought, whether they agreed or disagreed with each other, and why (or why not), and to fill the 5-min assignment time completely with out-loud discussion until the time was up, as indicated by the research assistants re-entering the room to give the next assignment.

**Table 1:** Description of the adapted sexuality-specific Peer Interaction Task (PIT)

Assignment	Description	Items
1. Plan your ideal party	Imagine, you may organize a party. There will be no parents or other adults present. Money is no issue and there are no rules for location or time. Discuss with each other how you want to organize this party and what you are going to do. Make a plan together: what would your ideal party look like?	
2. Boys versus girls	Look at the three cards about boys and girls, read them out loud. Discuss out loud what you think about each statement and why. Discuss out loud why you have the same or a different opinion	a. A boy should courtship a girl, not the other way around b. Girls should be less easy regarding sex than boys c. For a girl, it is more important to remain a virgin until marriage than for a boy
3. Condoms	Look at the three cards about condoms, read them out loud. Discuss out loud which statement fits you best and why. Discuss out loud why you have the same or a different opinion	a. I always use condoms during sex / I would always use condoms during sex, because… b. I never use condoms during sex / I would never use condoms during sex, because… c. I sometimes do and sometimes don’t use condoms during sex / I sometimes would and sometimes wouldn’t use condoms during sex, because…
4. Homosexuality	Look at the two cards about homosexuality, read and finish them out loud. Discuss out loud why you finish the sentences that way. Discuss out loud why you have the same or a different opinion	a. If a boy in my class would tell that he was gay, then… b. If a girl in my class would tell that she was a lesbian, then…
5. Boundaries and wishes	Look at the three cards about boundaries and wishes related to sex, read them out loud. Discuss out loud what you think about each statement and why. Discuss out loud why you have the same or a different opinion	a. A girl lets herself be courtshipped at first, but then doesn’t want sex after all. The guy pressures her a little to get sex anyway b. It isn’t bad to pressure someone a little if you want to have sex with him/her c. It can happen that you have sex with someone, even though you actually don’t want to, if he/she pressures you a little

#### Normativity and Deviance During Friendship Dyads’ Conversations About Sex

To assess the amounts of normativity and deviance during youths’ interactions with their friends while discussing sexuality-related topics, the content of the videotaped observations was transcribed verbatim and then coded. Two trained coders (one male and one female undergraduate and graduate student) independently coded the verbal interactions between the dyads (e.g., words or sentences) in the transcripts. Non-verbal interactions (i.e., facial expressions and body language) were coded while watching the videotapes. Each dyad member was coded separately. The coding manual used for the current study was based on the coding system for the original PIT (e.g., Dishion, Andrews, et al., 1995; Dishion et al., 1996; Patterson et al., 2000; Piehler & Dishion, 2007). To assess interrater reliability, ten randomly selected dyad members were coded by both coders. Interrater reliabilities were calculated with intraclass correlations, which ranged from *r*_ICC_ = .92 for deviant reinforcement to *r*_ICC_ = .97 for normative reinforcement and *r*_ICC_ = .98 for normative talk and deviant talk (all *p*s < .001). After obtaining these high interrater reliabilities, the remaining observations were coded by one of the two coders separately, but throughout the coding process, the coders regularly met to discuss their coding in order to prevent coding drift. In case of ambiguity or uncertainty about the coding, the coders always consulted with the main researchers.

##### Topic Codes

The coding system for the original PIT (e.g., Dishion et al., 1996, 2004) assessed two discussion topic codes: normative and rule-breaking talk. In the current study, the two topic codes that were used to evaluate the content of the dyadic interactions were “normative talk” and “deviant talk.”

*Normative talk* This code represented all instances of neutral talk, as well as talk that reflected healthy, positive, respectful, and tolerant sexuality-related attitudes and behaviors. In relation to the five assignments, this type of talk reflected reasonable partying; healthy, safe, pleasurable, and consensual sexual experiences for both partners; and equality of the sexes and of different sexual orientations. Examples of normative talk were: “It is not necessary that a boy has to come up to a girl to flirt with her, it can also be the other way around” [Dyad 31], “To pressure someone, that is something you just don’t do. When you have a bit of self-respect and respect for someone else, then you don’t do that” [Dyad 61].

*Deviant talk* This code covered all talks that qualified as non-normative and/or clearly deviant (i.e., reflecting risky, negative, disrespectful, and sexist or homophobic attitudes and behaviors). In relation to the five assignments, this type of talk reflected unreasonable partying (e.g., “A sort of Project X: break everything there is” [Dyad 9], “Mud wrestling for chicks” [Dyad 8]); unhealthy, unsafe, not pleasurable, and nonconsensual or disrespectful sexual experiences for at least one of the partners; and inequality of the sexes or of different sexual orientations. The latter included overt negativity toward homosexuality (e.g., that it is disgusting/not natural/should not be allowed), as well as stereotyped talk about gay guys (e.g., “If a boy in my class would tell that he was gay, I would give him a dildo for his birthday” [Dyad 8]), and hyper-sexualized talk about lesbian girls (e.g., “If a girl in my class would tell that she was a lesbian, I would find that kind of hot” [Dyad 8]). Other examples of deviant talk were “[Having sex] with a condom is so annoying. Then you have to interrupt, and then it’s like, oh wait, I have to go get condoms downstairs, or something” [Dyad 52], “I mean, when you go from one guy to the next, then you think like, yeah, that is a slut” [Dyad 58].

##### Reaction Codes

Besides topic codes, previous observation studies assessed various reaction codes to further evaluate the content of peer interactions, including laugh, pause, verbal assent, and nonverbal agreement (Dishion et al., 1996; Mathys et al., 2013; Piehler & Dishion, 2007). In the current study, two types of reaction codes were used: verbal reinforcement and nonverbal reinforcement. Verbal reinforcement included all instances of agreement (e.g., saying “yes”, “indeed”, “true”, etc.), and more or less literal repetition of what the friend had said before. Nonverbal reinforcement included smiling, laughing, nodding, thumbs-up, or “high-fives.” In this study, verbal and nonverbal reinforcements were combined (summed) into one reinforcement score. When reinforcement followed the friend’s normative talk, it was coded as *normative reinforcement*. When reinforcement followed the friend’s deviant talk, it was coded as *deviant reinforcement*.

After the coding was completed, count scores were calculated (i.e., summed) for each of the four codes (i.e., normative talk, deviant talk, normative reinforcement, deviant reinforcement), thus reflecting the number of times each code was given to each dyad member. Second, for each dyad member, relative scores for each of the four codes were computed as the proportion of the overall count scores (Patterson et al., 2000). Thus, the variables that were used in the analyses represented the amounts of normative and deviant talk and reinforcement relative to the overall coded interaction between each dyad member and his/her friend.

##### Perceived Sexual Peer Norms

Youths’ perceptions of sexual peer norms were measured in the online questionnaire. Four types of sexual peer norms were assessed: descriptive norms, injunctive norms, peer pressure, and risk norms.

*Descriptive norms* Youths’ perceptions of their friends’ sexual behaviors were measured with an item that is often used in the literature (e.g., Fasula & Miller, 2006): “How many of your best friends do you think have experience with intercourse?” (0 = *none of my friends*, 5 = *all of my friends*). A higher score indicated more sexually experienced friends.

*Injunctive norms* Youths’ perceptions of their friends’ sexual attitudes were measured with an adapted version of an item that has previously been used to measure parental sexual attitudes (e.g., Jaccard, Dittus, & Gordon, 1996): “My best friends believe that boys and girls our age should not yet have sex.” (0 = *completely not true*, 5 = *completely true*). Scores were reversed, so that a higher score indicated more approval of sexual activity from friends.

*Peer pressure* Experienced pressure from peers to have sex was measured with one item from the Peer Pressure Scale (e.g., Santor, Messervey, & Kusumakar, 2000): “I feel pressured to have sex, because a lot of people my own age have already had sex.” (0 = *never*, 5 = *very often*). Higher scores indicated more peer pressure to have sex.

*Risk norms* Youths’ perceptions of friends’ risky sexual behaviors were measured with two items (Deković et al., 2010): “How many of your best friends always use contraceptives (e.g., the pill or a condom) to prevent pregnancy when they have sex with someone?” and “How many of your best friends always use a condom to prevent STIs when they have sex with someone they cannot be sure does not have an STI?”^2^ (0 = *none of my friends*, 5 = *all of my friends*). Mean scores were computed of the two items, and scores were reversed, so that a higher score indicated more risky sexual behavior of friends.

### Data Analytic Strategy

Missing value analysis in SPSS version 22 indicated that 36.1–50.8 % of the sexual peer norm perception scores were missing, partly as a result of the fact that not all participants in the observation study completed the online questionnaire. Missing values were dealt with in two ways. For the descriptive analyses, Expectation–Maximization (Dempster, Laird, & Ruben, 1977) was used to estimate the missing values in SPSS version 22. In the APIM analyses, missing data were handled using Full Information Maximum Likelihood estimation in Mplus Version 7.3 (Muthén & Muthén, 2007). To account for non-normality in our data (a typical phenomenon in sex research), we used the Robust Maximum Likelihood (MLR) estimator, which corrects for deviation from multivariate normality by computing robust standard errors and an adjusted chi-square (Sass, Schmitt, & Marsh, 2014).

**Fig. 1:**
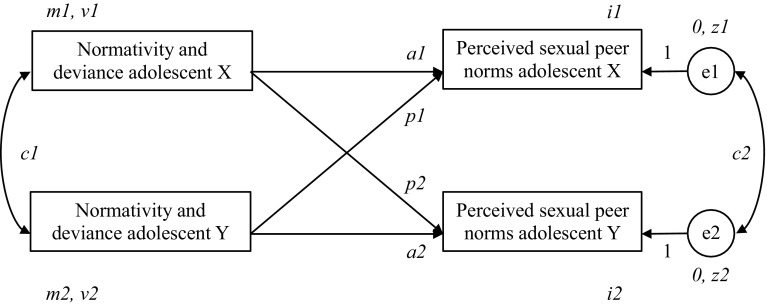
The actor–partner interdependence model (APIM) for perceived sexual peer norms predicted by the amounts of normativity and deviance during conversations about sex of indistinguishable friendship dyads. *Note.* The depicted parameters represent the actor effects (*a1* and *a2*), partner effects (*p1* and *p2*), predictor means (*m1* and *m2*), predictor variances *(v1* and *v2*), outcome intercepts (*i1* and *i2*), residual variances (*z1* and *z2*), and correlations between predictors (*c1*) and residual outcome variances (*c2*)

The data analysis consisted of two steps. First, the mean scores on the independent variables (normative and deviant talk and reinforcement) and the dependent variables (perceived sexual peer norms) were examined, and gender differences therein were assessed with *t*-tests. Second, the relations between dyadic normativity and deviance and youths’ individually perceived sexual peer norms were assessed with APIM (Olsen & Kenny, 2006) using structural equation modeling in Mplus Version 7.3. In APIM, path coefficients are estimated with the dyad as the unit of analysis. As the dyads in the current study were same-sex male or female dyads, they qualified as indistinguishable dyads, as opposed to distinguishable dyads such as, for instance, heterosexual couples (Mustanski, Starks, & Newcomb, 2014; Seiffge-Krenke & Burk, 2013). To estimate actor and partner effects for indistinguishable dyads, the models required six equality constraints, which give dyad members equal weight in the analyses (Olsen & Kenny, 2006). These are indicated in the caption under Fig. 1, which represents a depiction of the hypothesized actor–partner interdependence models.

In total, 16 APIMs were examined, each assessing the relation between a different combination of the independent variables (i.e., one of the four interaction codes) and the dependent variables (i.e., one of the four sexual peer norms). The APIM analyses were conducted in three steps. In the first step, unconstrained multigroup models were estimated, in which all paths were free to vary for male and female dyads. In the second step, the actor and partner effects in the multigroup models were individually constrained to be equal for male and female dyads. In the third step, chi-square difference tests were performed to assess whether these actor and partner effects differed significantly by gender. These tests involved a comparison of the fit of the constrained and unconstrained version of each model. Because the chi-square values for MLR cannot be used for chi-square difference testing in the typical manner, Satorra–Bentler-scaled (i.e., mean-adjusted) chi-square values were computed, which better approximate chi-square under non-normality by dividing the usual normal-theory chi-square statistic by a scaling correction (http://www.statmodel.com/chidiff.shtml). First, a difference test scaling correction, cd, was computed using the following formula: *cd* = (*d*_0_ * *c*_0_ − *d*_1_ * *c*_1_)/(*d*_0_ − *d*_1_), where *d*_0_ and *c*_0_ are the degrees of freedom and the scaling correction factor, respectively, for the null (i.e., constrained) model; and *d*_1_ and *c*_1_ are the degrees of freedom and the scaling correction factor for the comparison (i.e., unconstrained) model. Second, a Satorra–Bentler-scaled chi-square, TRd, was computed using the following formula: *TRd* = (*T*_0_ * *c*_0_ − *T*_1_ * *c*_1_)/*cd*, where *T*_0_ and *T*_1_ are the MLR chi-square values for the nested and comparison model, respectively. The resulting value was used for regular chi-square difference testing. A significantly higher chi-square value for the constrained model in comparison with the unconstrained model would indicate that the actor effects, the partner effects, or both, differed between boys and girls.

## Results

### Descriptive Analyses

Table 2 shows the means and *SD*s of the measures, separately for boys and girls. *t*-tests revealed no significant gender difference for normative talk. However, boys scored significantly higher on deviant talk than girls (medium-sized gender effect). Girls, on the other hand, reinforced normative talk significantly more often than boys (small to medium gender effect), but no significant gender difference was found in the reinforcement of deviant talk. Regarding perceived sexual peer norms, boys believed that their friends approved more of having sex (perceived injunctive norms) (medium to large gender effect), whereas girls believed that more of their friends engaged in risky sexual behavior (nearly medium gender effect). No significant gender differences were found in perceptions of how many of their friends had experience with sex (perceived descriptive norms) or experienced peer pressure to have sex.

**Table 2:** Ranges, means, SDs, and gender differences

	Range	Boys (*n* = 62) *M* (*SD*)	Girls (*n* = 60) *M* (*SD*)	*t*	df	*p*	*η* ^2^
Interaction codes							
Normative talk	23.08–64.21 %	41.53 % (7.48)	44.22 % (10.13)	−1.67	120	.098	.02
Normative reinforcement	6.07–48.34 %	20.44 % (7.20)	23.85 % (8.65)	−2.37	120	.019	.04
Deviant talk	5.19–33.33 %	19.99 % (5.39)	17.16 % (5.55)	2.86	120	.005	.06
Deviant reinforcement	0.75–29.06 %	10.06 % (4.59)	9.45 % (5.20)	0.70	120	.488	.00
Sexual peer norms							
Descriptive norms^a^	0–5	2.51 (1.06)	2.24 (0.99)	1.46	120	.146	.02
Injunctive norms^a^	0–5	4.62 (0.41)	4.09 (1.01)	3.82	120	<.001	.11
Peer pressure^a^	0–4	0.42 (0.69)	0.35 (0.48)	0.70	120	.484	.00
Risk norms^a^	0–5	1.12 (0.49)	1.45 (0.92)	−2.42	120	.018	.05

*η* ^2^ = Eta-squared effect size, with .01 = small effect, .06 = medium effect, and .14 = large effect (Cohen, 1988)

*t* = independent samples *t*-test statistic, *df* = degrees of freedom

^a^Absolute range, 0–5

### APIM Analyses

Table 3 shows the APIM results for the 16 tested models, separately for boys and girls.

**Table 3:** Standardized APIM estimates of perceived sexual peer norms predicted by the amounts of normativity and deviance during conversations about sex of indistinguishable male and female friendship dyads

		Descriptive norms			Injunctive norms			Peer pressure			Risk norms		
	C1	A	P	C2	A	P	C2	A	P	C2	A	P	C2
Male dyads (*k* = 31)													
Normative talk	.21	−.09	−.38***	.58***	−.15*	−.23**	−.27**	.01	−.07	.55***	−.30***	−.13	−.74***
Normative reinforcement	.19	−.35***	−.24*	.62***	.01	−.12	−.17*	.05	.12	.53***	−.10	−.09	.47
Deviant talk	.19	.46***	.32**	.51***	.09	.26*	−.23*	−.01	−.07	.54***	.29**	.19	.49
Deviant reinforcement	.16	.13*	.42***	.70***	.04	.10	−.17**	−.04	.07	.54***	.13	.01	.31
Female dyads (*k* = 30)													
Normative talk	.03	−.14	−.04	.51**	−.07**	.37***	.51***	.01	−.14	.23	−.26**	−.11	.49
Normative reinforcement	−.05	−.02	−.03	.48*	.00	−.06	.24	.08	.20*	.25	−.07	−.07	.47
Deviant talk	.24**	−.01	.06	.48*	.04	.11	.21	−.02	−.11	.23	.19*	.12	.39
Deviant reinforcement	.26*	.18*	−.11	.55***	.33***	−.37**	.49***	−.07	.11	.25	.09	.01	.51

C1-coefficients = within-dyad correlations between predictors, A = actor effects, P = partner effects, C2-coefficients = within-dyad correlations between residual outcome variances

* *p* < .05. ** *p* < .01. *** *p* < .001

#### Normative Talk

##### Descriptive Norms

In the first model, actor effects were similar for male and female dyads, Δ*χ* ^2^(1) = 1.60, *p* = .205. Both boys’ and girls’ own normative talk was not significantly related to their perceived descriptive norms. In the partner effects, a significant gender difference was found, Δ*χ* ^2^(1) = 10.81, *p* = .001. For boys, friends’ normative talk was related to perceiving fewer friends to be sexually experienced, whereas for girls there was no significant partner effect on perceived descriptive norms. The final model, in which actor effects were constrained and partner effects were freely estimated for boys and girls, explained 17 % of the variance in boys’ perceived descriptive norms (*R* ^2^ = .17, *p* = .035), but explained no significant variance in girls’ perceived descriptive norms (*R* ^2^ = .02, *p* = .493).

##### Injunctive Norms

In the second model, actor effects were similar for male and female dyads, Δ*χ* ^2^(1) = 2.35, *p* = .125. Both boys’ and girls’ own normative talk was related to perceiving less approval from friends to have sex. In the partner effects, a significant gender difference was found, Δ*χ* ^2^(1) = 5.83, *p* = .016. For boys, the partner effect had a similar direction (i.e., a negative sign) as the actor effect. Yet, for girls, a contrast pattern was observed: whereas girls’ own normative talk was related to perceiving less approval from friends of sexual behavior, their friends’ normative talk was related to perceiving more approval. The final model (actor effects constrained, partner effects freely estimated) explained 9 % of the variance in boys’ perceived injunctive norms (*R* ^2^ = .09, *p* = .030), and 14 % of the variance for girls (*R* ^2^ = .14, *p* = .006).

##### Peer Pressure

In the third model, actor and partner effects of normative talk on perceived peer pressure to have sex were similar for boys and girls, actor effects: Δ*χ* ^2^(1) = 0.17, *p* = .678, and partner effects: Δ*χ* ^2^(1) = 0.07, *p* = .789. Neither actor nor partner effects of normative talk on experienced peer pressure were significant. Consistent with these nonsignificant effects, the final model (actor and partner effects constrained) explained no significant variance in experienced peer pressure for boys (*R* ^2^ = .01, *p* = .472) or girls (*R* ^2^ = .02, *p* = .385).

##### Risk Norms

In the fourth model, actor and partner effects of normative talk on perceived risk norms were similar for boys and girls, actor effects: Δ*χ* ^2^(1) = 0.00, *p* = .997, and partner effects: Δ*χ* ^2^(1) = 0.28, *p* = .594. Whereas boys’ and girls’ own normative talk was related to perceiving fewer friends to engage in risky sexual behavior, partner effects of normative talk were not significantly related to perceived risk norms. The final model (actor and partner effects constrained) explained significant variance (12 %) in boys’ perceived risk norms (*R* ^2^ = .12, *p* = .030), but not for girls (*R* ^2^ = .08, *p* = .076).

#### Normative Reinforcement

##### Descriptive Norms

In the first model, significant gender differences were found in both actor, Δ*χ* ^2^(1) = 7.34, *p* = .007, and partner effects, Δ*χ* ^2^(1) = 4.77, *p* = .029. Both boys’ own normative reinforcement and that of their friend was related to perceiving fewer friends to be sexually experienced, whereas for girls there were no significant actor or partner effects on perceived descriptive norms. The final model, where actor and partner effects were freely estimated for boys and girls, explained 21 % of the variance in boys’ perceived descriptive norms (*R* ^2^ = .21, *p* = .037), but explained no significant variance for girls (*R* ^2^ = .00, *p* = .899).

##### Injunctive Norms

In the second model, no significant gender differences were found in the actor, Δ*χ* ^2^(1) = 2.94, *p* = .086, or partner effects, Δ*χ* ^2^(1) = 2.81, *p* = .094. For both male and female dyads, neither actor nor partner effects of normative reinforcement were significant. The final model (actor and partner effects constrained) explained no significant variance in perceived injunctive norms for boys (*R* ^2^ = .01, *p* = .656) or girls (*R* ^2^ = .00, *p* = .696).

##### Peer Pressure

In the third model, actor and partner effects of normative reinforcement on perceived peer pressure to have sex were similar for boys and girls, actor effects: Δ*χ* ^2^(1) = 2.40, *p* = .121, and partner effects: Δ*χ* ^2^(1) = 1.05, *p* = .306. Although we observed that for female dyads, friends’ reinforcement of normative talk was significantly related to experiencing more peer pressure to have sex, the final model (actor and partner effects constrained) explained no significant variance in experienced peer pressure for boys (*R* ^2^ = .02, *p* = .454) or girls (*R* ^2^ = .04, *p* = .387).

##### Risk Norms

In the fourth model, actor and partner effects of normative reinforcement on perceived risk norms were similar for boys and girls, actor effects: Δ*χ* ^2^(1) = 1.27, *p* = .260, and partner effects: Δ*χ* ^2^(1) = 2.64, *p* = .104. Neither actor effects nor partner effects were significantly related to boys’ and girls’ perceptions of their peers’ sexual risk behavior. Consistent with these nonsignificant effects, the final model (actor and partner effects constrained) explained no significant variance in perceived risk norms of boys (*R* ^2^ = .02, *p* = .543) or girls (*R* ^2^ = .01, *p* = .576).

#### Deviant Talk

##### Descriptive Norms

In the first model, significant gender differences were found in both the actor, Δ*χ* ^2^(1) = 7.96, *p* = .005, and partner effects, Δ*χ* ^2^(1) = 6.45, *p* = .011. Both boys’ own deviant talk and that of their friend was related to perceiving more friends to be sexually experienced, whereas for girls actor and partner effects on perceived descriptive norms were not significant. The final model, in which actor and partner effects were freely estimated, explained 37 % of the variance in boys’ perceived descriptive norms (*R* ^2^ = .37, *p* < .001), but explained no significant variance for girls (*R* ^2^ = .00, *p* = .759).

##### Injunctive Norms

In the second model, no significant gender differences were found in actor, Δ*χ* ^2^(1) = 0.63, *p* = .426, or partner effects, Δ*χ* ^2^(1) = 1.00, *p* = .318. Yet, whereas for female dyads neither actor effects nor partner effects of deviant talk on perceived injunctive norms were significant, for male dyads, friends’ deviant talk was related to perceiving more approval from friends to have sex. However, the final model (actor and partner effects constrained) explained no significant variance in perceived injunctive norms for boys (*R* ^2^ = .09, *p* = .137) or girls (*R* ^2^ = .01, *p* = .286).

##### Peer Pressure

In the third model, actor and partner effects of deviant talk on perceived peer pressure to have sex were similar for boys and girls, actor effects: Δ*χ* ^2^(1) = 2.00, *p* = .158, and partner effects: Δ*χ* ^2^(1) = 0.50, *p* = .480. Neither actor nor partner effects of deviant talk were significant. Consistent with these nonsignificant effects, the final model (actor and partner effects constrained) explained no significant variance in experienced peer pressure for boys (*R* ^2^ = .01, *p* = .657) or girls (*R* ^2^ = .01, *p* = .670).

##### Risk Norms

In the fourth model, actor and partner effects of deviant reinforcement on perceived risk norms were similar for boys and girls, actor effects: Δ*χ* ^2^(1) = −0.93, *p* = .336, and partner effects: Δ*χ* ^2^(1) = −0.28, *p* = .594. Whereas boys’ and girls’ own deviant talk was related to perceiving more friends to engage in risky sexual behavior, partner effects of deviant talk were not significant. The final model (actor and partner effects constrained) explained significant variance (14 %) in boys’ perceived risk norms (*R* ^2^ = .14, *p* = .016), but not for girls (*R* ^2^ = .06, *p* = .138).

#### Deviant Reinforcement

##### Descriptive Norms

In the first model, actor effects were similar for male and female dyads, Δ*χ* ^2^(1) = 0.94, *p* = .332. For both boys and girls, own deviant reinforcement was related to perceiving more friends to be sexually experienced. A significant gender difference was found in the partner effects, Δ*χ* ^2^(1) = 8.44, *p* = .004. Boys’ friends’ deviant reinforcement was related to perceiving more friends to be sexually experienced, whereas for girls the partner effect was not significant. The final model, where actor effects were constrained and partner effects were freely estimated, explained 21 % of the variance in boys’ perceived descriptive norms (*R* ^2^ = .21, *p* = .002), but explained no significant variance for girls (*R* ^2^ = .03, *p* = .314).

##### Injunctive Norms

In the second model, significant gender differences were found in both actor, Δ*χ* ^2^(1) = 4.52, *p* = .034, and partner effects, Δ*χ* ^2^(1) = 6.83, *p* = .009. For boys, actor and partner effects of deviant reinforcement were not significant. For girls, another contrast pattern was found: whereas girls’ own deviant reinforcement was related to perceiving more approval from friends of sexual behavior, their friends’ deviant reinforcement was related to perceiving less approval. The final model (actor and partner effects freely estimated) explained no significant variance for boys (*R* ^2^ = .01, *p* = .468), but explained 19 % of the variance in girls’ perceived injunctive norms (*R* ^2^ = .19, *p* = .049).

##### Peer Pressure

In the third model, actor and partner effects of deviant reinforcement on perceived peer pressure to have sex were similar for boys and girls, actor effects: Δ*χ* ^2^(1) = 0.26, *p* = .610, and partner effects: Δ*χ* ^2^(1) = 2.87, *p* = .091. Neither actor nor partner effects were significant. Consistent with these nonsignificant effects, the final model (actor and partner effects constrained) explained no significant variance in experienced peer pressure for boys (*R* ^2^ = .01, *p* = .384) or girls (*R* ^2^ = .01, *p* = .499).

##### Risk Norms

In the fourth model, actor and partner effects of deviant reinforcement on perceived risk norms were similar for boys and girls, actor effects: Δ*χ* ^2^(1) = 1.35, *p* = .245, and partner effects: Δ*χ* ^2^(1) = 0.09, *p* = .763. Neither actor effects nor partner effects were significantly related to boys’ and girls’ perceptions of their friends’ risky sexual behavior. Consistent with these nonsignificant effects, the final model (actor and partner effects constrained) explained no significant variance in perceived risk norms of boys (*R* ^2^ = .02, *p* = .582) or girls (*R* ^2^ = .01, *p* = .629).

## Discussion

Although the relation between youths’ perceptions of existing sexual norms among peers and their own sexual behaviors has been well established (e.g., van de Bongardt et al., 2015), the micro-time processes (e.g., dyadic interactions over the course of seconds, minutes, or hours) (Patterson et al., 2000; Wachs, 2015) through which youth and their peers might interactively construct such norms remain less well understood. The current study was the first to examine how conversations about sex (and specifically—normativity and deviance) during observed dyadic interactions with same-sex friends were related to adolescents’ and young adults’ individual perceptions of sexual peer norms.

First, the results of the APIM (Olsen & Kenny, 2006) analyses showed that the amounts of normativity and deviance during sexual communication with friends were indeed related to perceived descriptive, injunctive, and risky sexual peer norms. As hypothesized (*H1a*), overall, more normative talk and reinforcement thereof was related to youths’ perceptions that fewer friends had experience with sexual behaviors or had engaged in risky sexual behavior, and that their friends approved less of having sex. In contrast, more deviant talk and reinforcement thereof was related to youth perceiving that more friends had experience with sexual behaviors or had engaged in risky sex, and that their friends were more approving of having sex (*H1b*).

Unexpectedly, observed normativity and deviance during sexual communication with friends were overall not significantly related to youths’ experienced peer pressure to have sex. This may be explained by the fact that the participants in the current study reported very little pressure from peers to have sex. On the one hand, this might confirm that adolescents and young adults generally experience substantial agency in their behavioral decisions, including those related to sex (Ungar, 2000). On the other hand, it may be an indication of the difficulty for (young) people to recognize and acknowledge external social pressure when making behavioral decisions, and their susceptibility to it.

Another possible explanation may lie in the fact that the majority of the observed interactions was between good or best friends, who may not exert overt and direct pressure on each other to have sex. In the recent meta-analysis of van de Bongardt et al. (2015), it was found that adolescent sexual behavior was more strongly associated with peer pressure of more distant peers (i.e., peers in general) than of closer friends. This suggests that conformity to sexual peer pressure might be a way to become accepted by more distant, high-status peers (Brechwald & Prinstein, 2011). Yet, overall, this meta-analysis showed that peer pressure was least strongly related to adolescent sexual behavior in comparison with descriptive and injunctive norms. Thus, although peer pressure is often considered an important predictor of youth sexual behavior, its conceptualization as an overt and direct form of social influence might underestimate the complexity of the sociopsychological processes that underlie the mechanism of peer pressure, which may, in fact, operate more subtly, indirectly, and unconsciously. In future studies, researchers should reconsider how evidence of peer pressure can be reliably and validly measured. Instead of relying on self-reports, experimental study designs may prove to be a promising alternative (see e.g., Choukas-Bradley, Giletta, Widman, Cohen, & Prinstein, 2014; Widman, Choukas-Bradley, Helms, & Prinstein, 2016).

Second, APIM results revealed that perceptions of descriptive and injunctive norms were both related to the amounts of adolescents’ and young adults’ own normativity and deviance (actor effects), as well as to their friends’ normativity and deviance (partner effects) during the observed peer interactions. However, risk norm perceptions were only related to youths’ own normative and deviant talk (i.e., actor effects only). Thus, hypothesis *H2* was partially confirmed, which stresses the importance of acknowledging the contribution of both dyad members as *targets* and *sources* of mutual social influence in peer interactions. By combining an observational and dyadic research approach, the current study bridged the gap between the literature on observed deviancy training processes where typically one target per dyad is selected for the analyses (Capaldi et al., 2001; Dishion, Andrews, et al., 1995, Dishion et al., 1996, 1997, 2004; Patterson et al., 2000; Piehler & Dishion, 2007), and studies on dyadic peer interactions and relations which typically rely on self-reports (Cillessen, Jiang, West, & Laszkowski, 2005; Giletta et al., 2011). This combined approach may further advance research in both fields.

Third, several gender differences were found in the mean scores on the assessed variables. In line with our expectations and previous research (Dishion, 2000; Piehler & Dishion, 2007), boys scored higher on deviant talk, whereas girls more often reinforced normative talk. No significant differences were found, however, in the amounts of normative talk and deviant reinforcement. Thus, hypothesis *H3a* was only partially confirmed. With respect to sexual peer norm perceptions, boys believed that their friends approved more of having sex (perceived injunctive norms), whereas girls believed that more of their friends engaged in risky sexual behavior (perceived risk norms). This is partially in line with our expectations and with empirical findings that show the still existing sexual double standard (Crawford & Popp, 2003; Kreager & Staff, 2009; Lyons et al., 2011). The unexpected finding of girls reporting more sexually risk-taking friends might be explained by the relatively high proportion of other-sex friends in girls’ peer networks (Boislard & Poulin, 2011), and girls’ greater tendency to have older male friends (Poulin, Denault, & Pedersen, 2011). However, as no significant differences were found in perceived descriptive norms and peer pressure, hypothesis *H3b* was also only partially confirmed.

Besides these gender differences in mean scores, the APIM results also revealed different patterns in the investigated relations for male and female dyads (*H3c*). In short, whereas, for boys, the amounts of normativity and deviance during sexual communication with friends was related to their perceptions of three types of sexual peer norms (i.e., descriptive, injunctive, and risk norms), for girls, it was associated only with injunctive norms. Finding stronger links between sexual communication with friends and perceived sexual peer norms for boys matches with notions that sexual behaviors (and talking about those behaviors) are important for boys’ same-sex peer acceptance (Kreager & Staff, 2009; Reed & Weinberg, 1984). The fact that we found a diverging gendered pattern in comparison with the study of Busse et al. (2010), who found that for girls, but not for boys, more frequent sexual communication with friends was associated with perceiving more pressure (i.e., a combination of descriptive and injunctive norms) to have sex, emphasizes the importance of making a distinction between the different types of sexual peer norms, as well as between how often youths talk about sexuality-related topics with their friends, and the way in which they talk about such topics (i.e., how normatively or deviantly).

### Strengths, Limitations, and Directions for Future Research

In comparison with previous observation studies that examined only general and not sexuality-specific peer interactions (for an exception, see Capaldi et al., 2001), that selected only one target per dyad for the analyses (e.g., Capaldi et al., 2001; Dishion et al., 2004; Patterson et al., 2000; Piehler & Dishion, 2007), and that typically included only male dyads (for exceptions, see Dishion, 2000; Piehler & Dishion, 2007), the current study had several major strengths. It was the first study to observe friendship dyads performing a peer interaction task that focused specifically on sexual communication, and the first study to investigate how the amounts of normativity and deviance during these peer interactions were related to youths’ individual perceptions of sexual peer norms. Furthermore, by performing APIM analyses (Olsen & Kenny, 2006), we more optimally utilized the dyadic nature of these peer interactions. Finally, observing both male and females dyads allowed us to compare sexuality-specific peer interactions between boys and girls, and to assess gender differences in the links between these interactions and individual perceptions of sexual peer norms. A better understanding of gender differences and similarities is valuable for the improvement of gender-sensitive prevention and intervention practices that aim to reduce youth’s susceptibility to potentially risky peer influences on their sexual health.

Despite these strengths, several limitations should also be discussed. First, as the current study had a cross-sectional design, no conclusions can be drawn about the temporal relation of the observed normativity and deviance during sexual communication and individual perceptions of sexual peer norms. Thus, although we considered communication styles as predictors of sexual peer norm perceptions in the analyses, we were not able to assess whether the peer interactions affected youths’ individual perceptions of sexual norms, or whether these individual perceptions of sexual peer norms may have affected the way in which youth talked about sex with their friends. Most likely, these relation are bidirectional, and both processes play a role. Longitudinal and experimental research designs are needed to assess this further.

Second, the sexual peer norms were measured with one-item instruments, which is common practice in research on the relation between sexual peer norms and youth sexual behavior (for a review, see the meta-analysis of van de Bongardt et al., 2015). Although single-item measures do not possess optimal psychometric quality, studies that have investigated the use of single-item versus multiple-item measures for various constructs (e.g., self-reported attitudes, beliefs, perceptions, or health-related quality of life) have found that neither method appears to be empirically better than the other (Cunny & Perri, 1991; Gardner, Cummings, Dunham, & Pierce, 1998). Nonetheless, one-item instruments may not fully capture the complexity of peer influence processes. Experimental research, for instance, might further improve our understanding of the complex sociopsychological processes that underlie the mechanisms through which interactions with peers affect youths’ individual perceptions of sexual peer norms, and how these, in turn, affect their sexual decision making.

Third, although our sample included 19 % ethnic minority youth, this group was too small and too diverse to analyze ethnic differences. Based on research showing that youth with more collectivist (non-Western) ethnic backgrounds tend to be more susceptible to the influence of their friends than youth with more individualistic (Western) ethnic backgrounds (Verkuyten & Masson, 1996), it may be expected that the relationship between sexual communication with friends and individual perceptions of sexual peer norms are stronger for non-Western youth. Insight into ethnic differences, through replication with more ethnically diverse samples, or samples drawn from cultures in which emerging sexual activity during adolescence is not considered normative, is paramount for the improvement of culturally sensitive sexual health promotion programs aimed at reducing potentially risky peer influences.

Finally, together, the findings of the current study and the previous study of Busse et al. (2010) suggest that both investigated aspects of sexual communication between friends (i.e., frequency and manner) relate to youths’ individual perceptions of sexual peer norms in different ways. Yet, these two aspects are also likely to be linked: in line with the notions of observational learning (Bandura, 1971), it can be expected that the effects of normative or deviant sexual communication with friends on individually perceived sexual norms, attitudes, and behaviors will depend on how frequently conversations about sex take place. In the present study, we did not assess how often the participating friendship dyads normally (i.e., outside of the peer interaction task) talked about sexuality-related topics. Investigating this further, for instance, by collecting both micro-time observational data on sexual communication styles and macro-time longitudinal questionnaire data on sexual communication frequency among a sample of friendship dyads, is a pertinent direction for future research.

### Conclusions

Notwithstanding these limitations, the findings of the current study contribute to the theoretical understanding of how relations with peers may affect youths’ behaviors, by demonstrating that the way in which adolescents and young adults talk about sexuality-related topics with their friends (i.e., the amounts of normativity and deviance) relates to their individual perceptions of peer norms regarding sexual behavior, which, in turn, have been found to be related to adolescents’ actual sexual behavior (van de Bongardt et al., 2015). Our results shed new light on a potential mechanism (i.e., perceptions of behavioral norms among peers) that may explain the consistently identified association between micro-time processes of deviancy training during peer interactions and the macro-time development of various problem behaviors during adolescence and young adulthood (e.g., Dishion, Capaldi, et al., 1995, Dishion et al., 1997; Patterson et al., 2000). However, more research is needed to examine whether this mechanism applies to other behaviors as well. It is noteworthy that the hypothesized associations between the amounts of observed normativity and deviance and perceived descriptive and injunctive norms were found among the current study sample of 16–21-year-old adolescents and young adults. At this age, sexual behavior can be considered normative (De Graaf et al., 2012; Tolman & McClelland, 2011), and thus perceiving friends as sexually active or as approving thereof would not necessarily be problematic. An important next step, therefore, is to replicate this research with younger adolescents, for whom this mechanism and its outcomes might be more problematic. Besides possible age differences, more generally it is important to further investigate which youths are most susceptible to potentially risky effects of deviant interactions with peers. This, too, is an important direction for future research.

## Footnotes

1 The full protocol that was used for the observations of sexuality-specific interactions within friendship dyads is available from the corresponding author.

2 This item was accompanied by a note explaining the meaning of STIs: “STIs are sexually transmitted infections, also called “genital diseases.” These are diseases you can get by having unprotected sex. Examples are chlamydia, gonorrhea, syphilis, herpes, or HIV: the virus that causes AIDS.”
